# Supplementary material for: Antisense oligonucleotide activity in tumour cells is influenced by intracellular LBPA distribution and extracellular vesicle recycling
Source: Commun Biol. 2021 Nov 1;4:1241. doi: 10.1038/s42003-021-02772-0 (PMC8560811; doi:10.1038/s42003-021-02772-0)
Supplement: Supplementary file 2 — Supplementary information [file 42003_2021_2772_MOESM2_ESM.pdf]

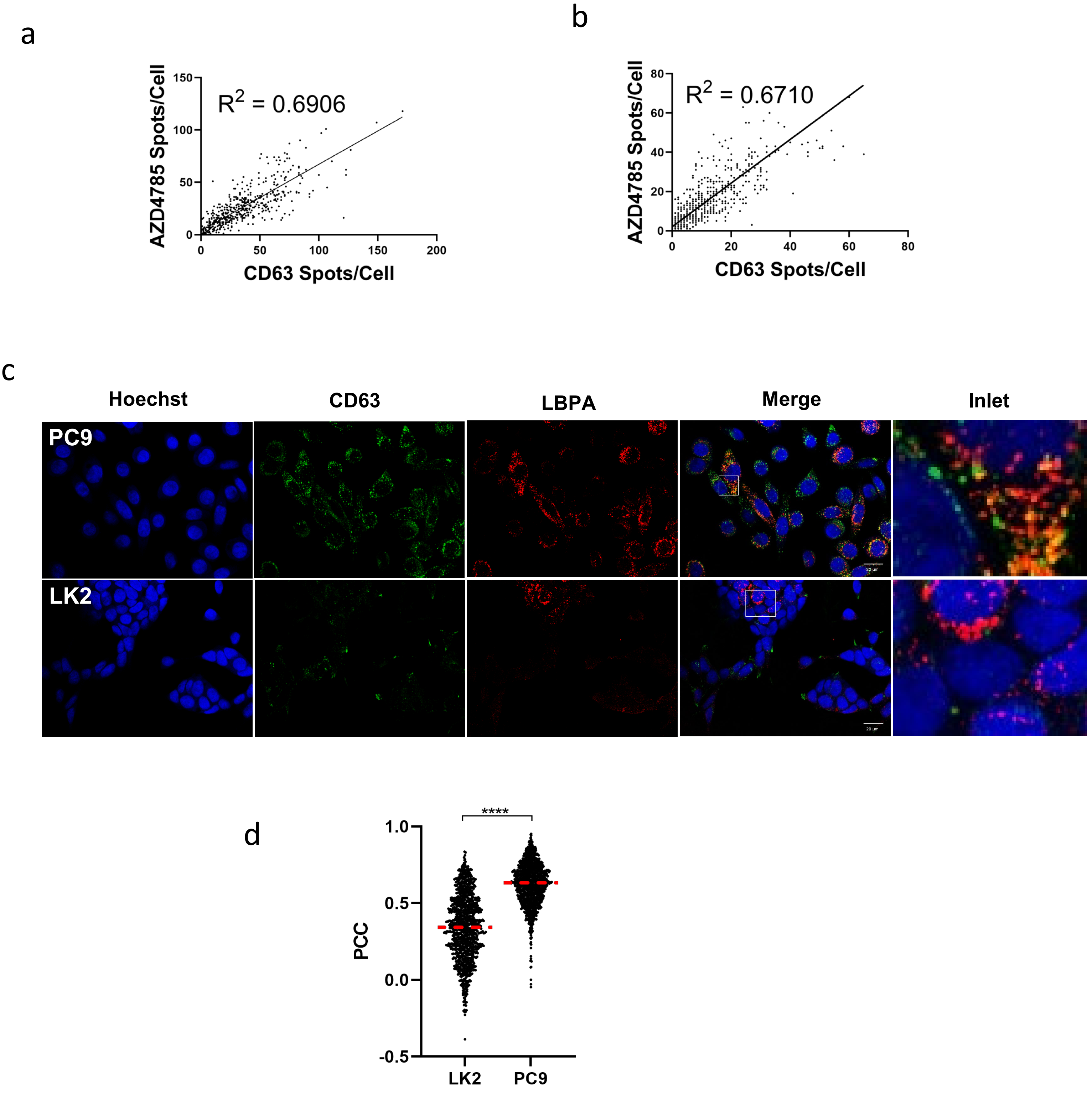

Supplementary Figure 1. AZD4785 endocytosis level correlates with CD63 but not LBPA.

a, b PC9 and LK2 cells were incubated with AZD4785 for 6h, then fixed and stained. LE and AZD4785 were stained with anti-CD63 and anti-PS-ASO antibodies, respectively and visualized using secondary fluorescently-labelled antibodies. Nuclei were stained with Hoechst33342. Cell images were acquired using Opera microscope (60x objective, NA1.4). Intracellular content of AZD4785 and CD63 (Spots/Cell) was quantified using Columbus software. **a** Correlation between number of spots of AZD4785 and CD63 in single PC9 cells. N=3, n = 496. Linear regression analysis. **b** Correlation between number of spots of AZD4785 and CD63 in single LK2 cells. N=3, n = 582. Linear regression analysis. **c, d** CD63 and LBPA cellular distribution in PC9 and LK2 cells. Cells were stained with anti-CD63 and anti-LBPA antibodies and visualized using secondary fluorescently-labelled antibodies. Nuclei were stained with Hoechst33342. Cell images were acquired using Opera microscope (40x objective, NA1.4). CD63 and LBPA colocalization was measured by Pearson's correlation coefficient (PCC) by using Columbus software . N=3, Median, red dotted line, n (LK2) = 1172, n (PC9) = 1551, ****, P< 0.0001, t test.

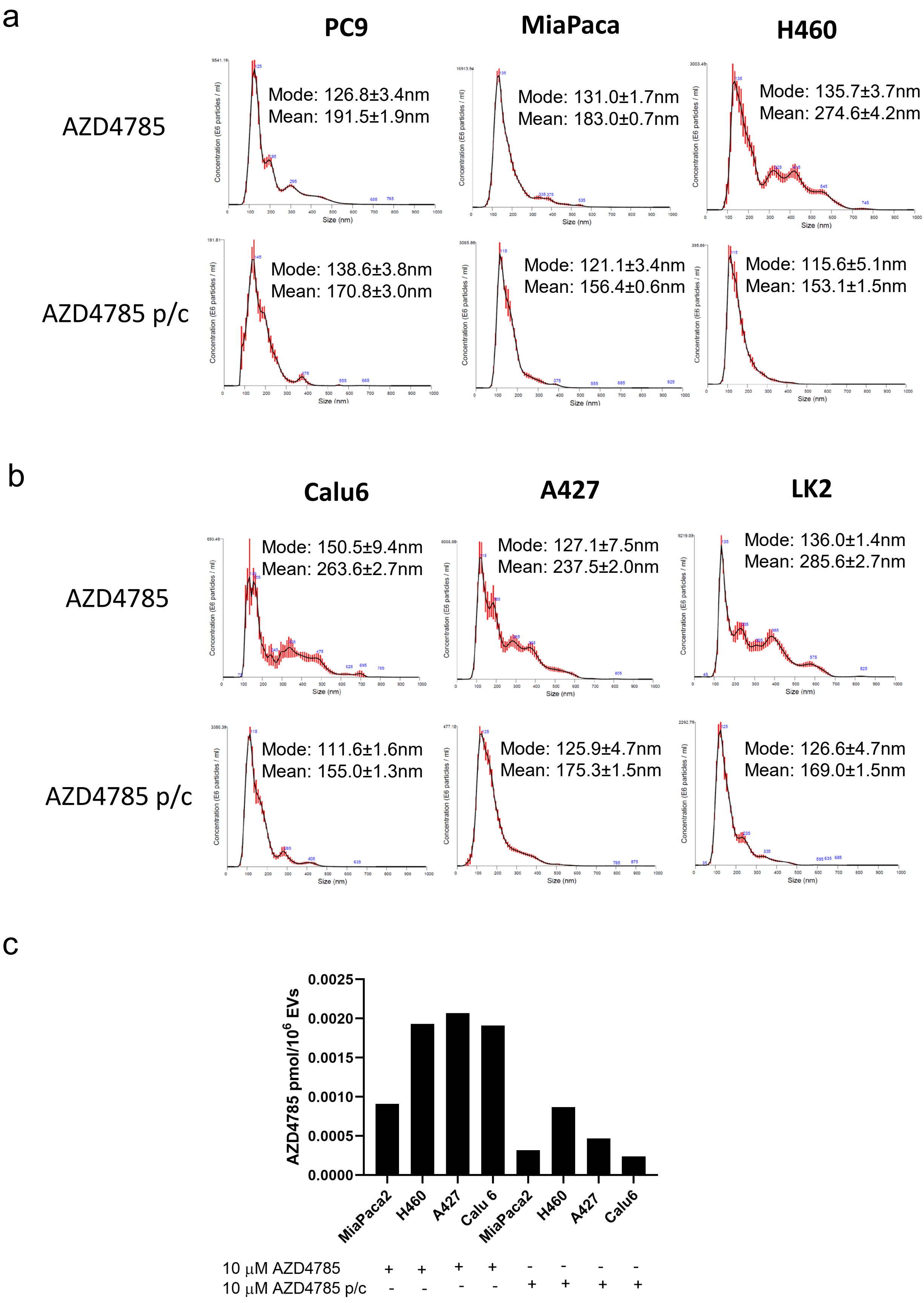

Supplementary Figure 2. AZD4785 modulates EV secretion by tumour cells.

Good (**a**) and poor (**b**) productive PS-ASO uptake tumour cells were treated with 10µM AZD4785 ("AZD4785") for 24h. For pulse-chase experiment, cells were treated with 10µM AZD4785 for 6h, washed and incubated for 24h ("AZD4785 p/c"). EVs were isolated from conditioned cell media by differential ultracentrifugation and a 100,000x*g* pellets were analysed by NTA. Representative NTA images. (N=2-6) **c** Quantification of AZD4785 in isolated EVs using quantitative mass spectrometry. EVs isolated as in (**a**) were analysed by UPLC-MS. Representative data from N=2.

a

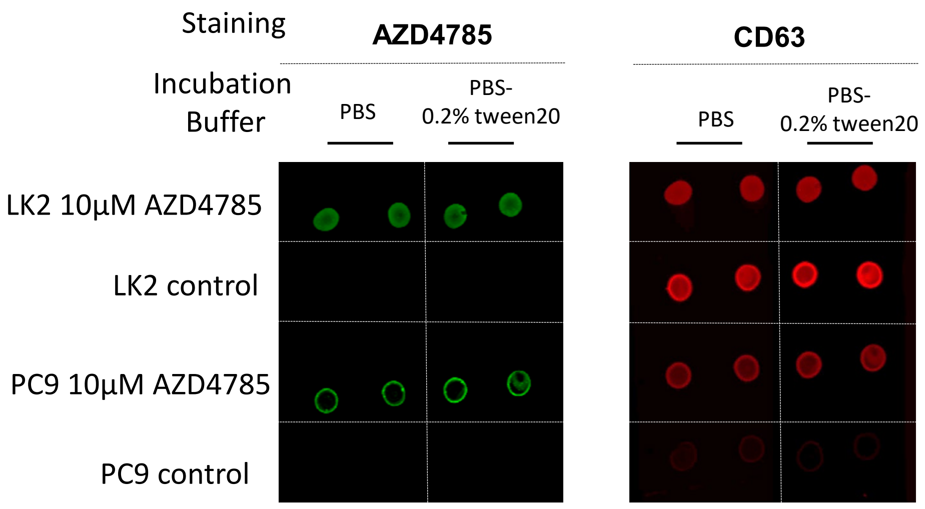

b

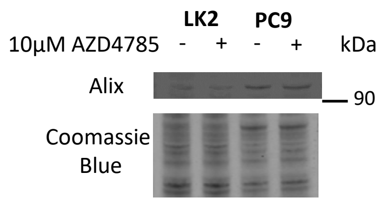

c

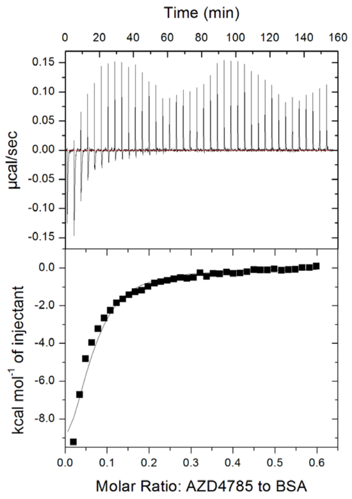

K_d (µM)	ΔH (kcal/mol)	ΔG (kcal/mol)	-TΔS (kcal/mol)
8.18± 0.52	-13.3± 0.06	-6.72± 0.32	6.62± 0.38

Supplementary Figure 3. AZD4785 is secreted in EV by PC9 and LK2 cells. a AZD4785, Alix and CD63 secretion in EVs as detected by the dot-blot. PC9 and LK2 were treated with 10 µM AZD4785 for 24h and EVs were isolated from conditioned media. An aliquot was applied to the nitrocellulose membrane, air-dried and stained with anti-ASO, anti-Alix or anti-CD63 antibody in the absence or presence of permeabilising detergent, tween-20 (0.2%). Membrane was visualised with Odyssey ® CLx Infrared Imaging System. Each experiment was conducted in duplicate. Representative from N=3. **b** Detection of Alix in the whole cell lysates. PC9 and LK2 cells were treated with or without AZD4785 for 24h and lysed in RIPA buffer. Equal amounts of whole cell lysate (10µg) were analysed by western blotting and probed for Alix. Representative image from N=2. **c** Albumin interaction with AZD4785 measured by isothermal titration calorimetry (ITC). Binding affinity was measured in PBS (pH 7.4), 10mM PIPES (pH 7.4) containing 140mM NaCl and 2.5mM $CaCl_2$. AZD4785 (870 µM final concentration) was titrated into a BSA solution (200 µM). The latter was directly prepared for dissolution in a mixture of the buffer:water 30:1 to match the AZD4785 conditions. The data were collected at 25°C, using 1 µL sequential injections every 4min. Background level was derived from heat of dilution of AZD4785 in the buffer and subtracted from the experimental values. Data were analysed using the ‘One set of sites’ model function in the Origin 7 software, which defines that all binding sites are equal. Each ITC experiment was performed in duplicates and data reported are averaged. Since multiple BSA seemed to bind onto one single ASO strand with, possibly, different types of interactions affinities, the stoichiometry was fixed to a 0.06 AZD4785 : BSA ratio for all data analyses. This value was chosen as it corresponds to 1 BSA molecule for nucleotide and it allows for a reasonable good fitting. Since it was not possible to obtain the actual binding stoichiometry the calculated affinity values have to be considered as a whole system value. Note that multiple albumin molecules bind to the molecule of ASO but molar ratio was limited to albumin:ASO = 1:16 to reflect 16mer nature of AZD4785 and to achieve the best ITC curve fit.

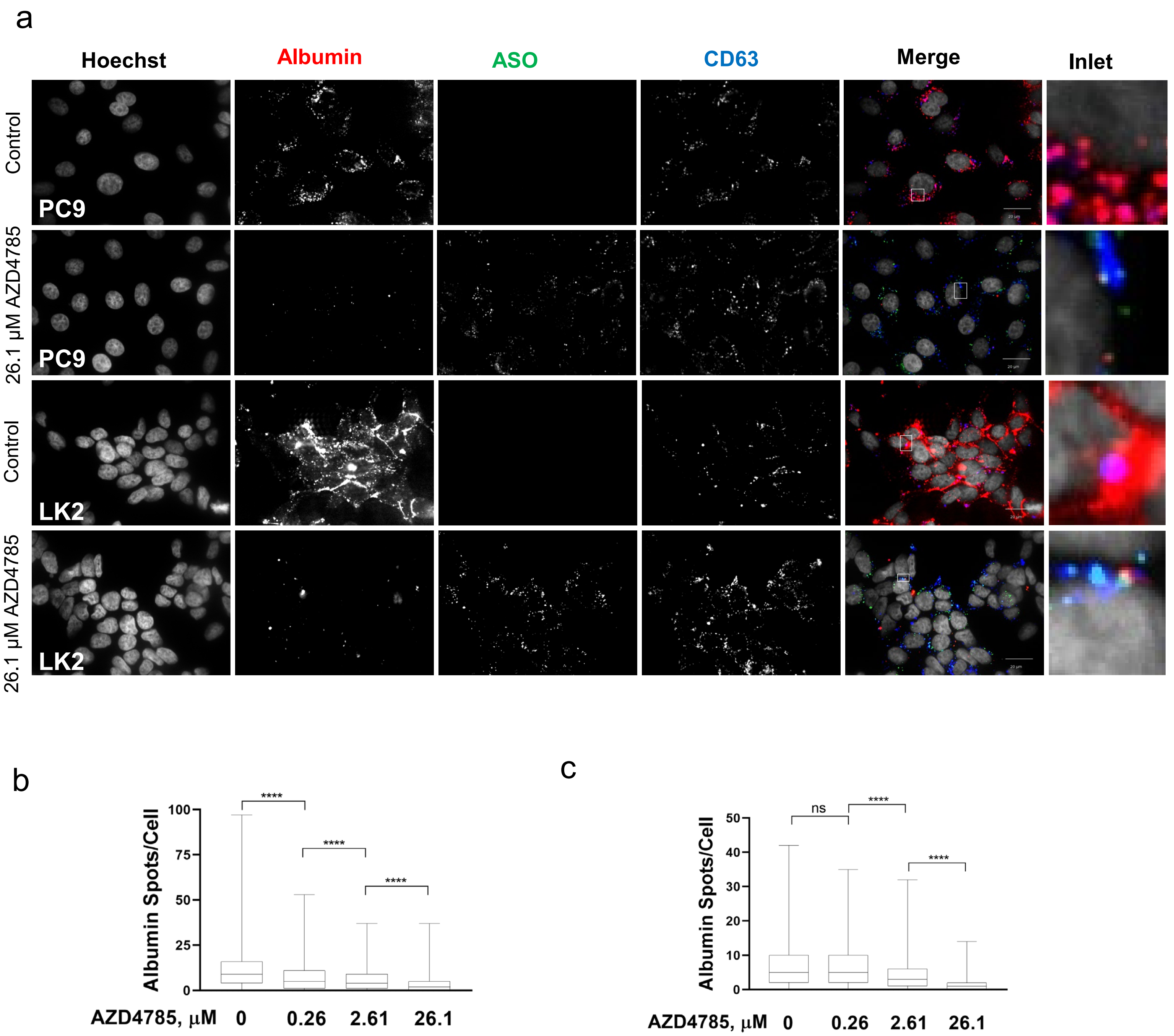

Supplementary Figure 4. AZD4785 reduces albumin endocytosis by PC9 and LK2 cells.

PC9 cells (**a** and **b**) and LK2 cells (**a** and **c**) were incubated in low serum media (RMPI-0.5%FBS) for 24h and AZD4785 and/or Alexa594-labelled albumin (100µg/ml) were added to cells for 3h. Cells were fixed, permeabilised and stained for ASO, CD63 and nucleus. Cell images were acquired using Opera microscope (60x objective, NA1.4) Intracellular content of AZD4785, albumin-Alexa594 and CD63 was quantified using Columbus software by counting number of stained spots in the cell cytosol ("Spots/Cell") (N=3, n=586-989). Whiskers, Min to Max. ANOVA ****, p < 0.0001. **, p<0.01. ns, non-significant.

a

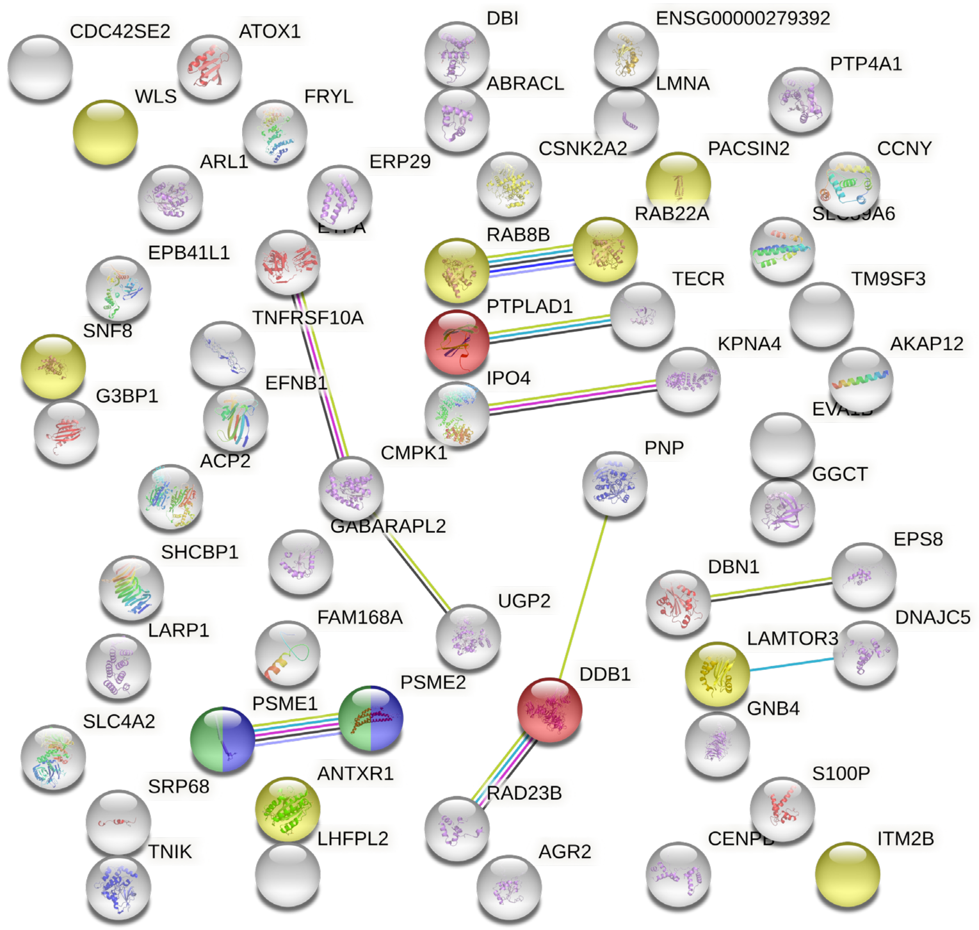

b

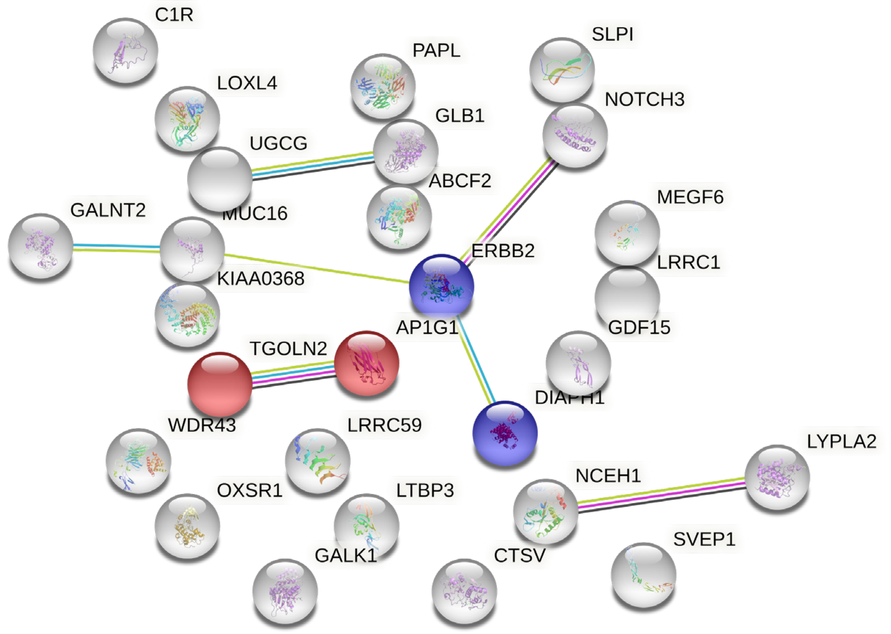

Supplementary Figure 5. STRING analysis of protein enrichment in EVs isolated from PC9 cell.

a Proteins exclusively presented in PC9-derived EVs (n = 58) were analysed by STRING database. 57 nodes/10 edges were identified. Nodes with top function enrichment are highlighted with red (Biological Process (Gene Ontology), GO:0046726, “positive regulation by virus of viral protein level in host cell, strength 2.23), blue (Molecular Function (Gene Ontology), GO:0061133, endopeptidase activator activity, strength 2.06), green (Cellular Component (Gene Ontology), GO:0008537, proteasome activator complex, strength 2.36) and yellow (Cellular Component (Gene Ontology), GO:0010008, endosome membrane, strength 0.0046).

b Proteins exclusively presented in EVs isolated from AZD4785-treated PC9 cells (n = 28) were analysed by STRING database. 27 nodes/7 edges were identified. Nodes with top function enrichment are highlighted with red (Cellular Component (Gene Ontology), GO:0030140, trans-Golgi network transport vesicle, strength 1.71) and blue (Reactome Pathways, HSA-6785631, ERBB2 Regulates Cell Motility, strength 2.05).

a

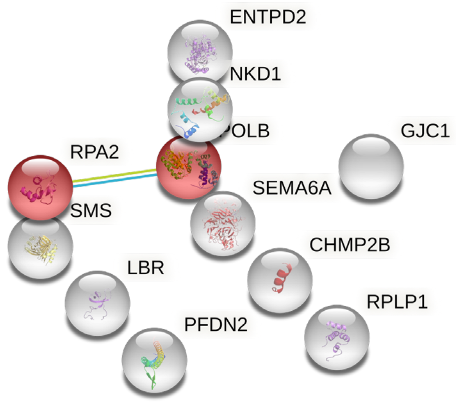

b

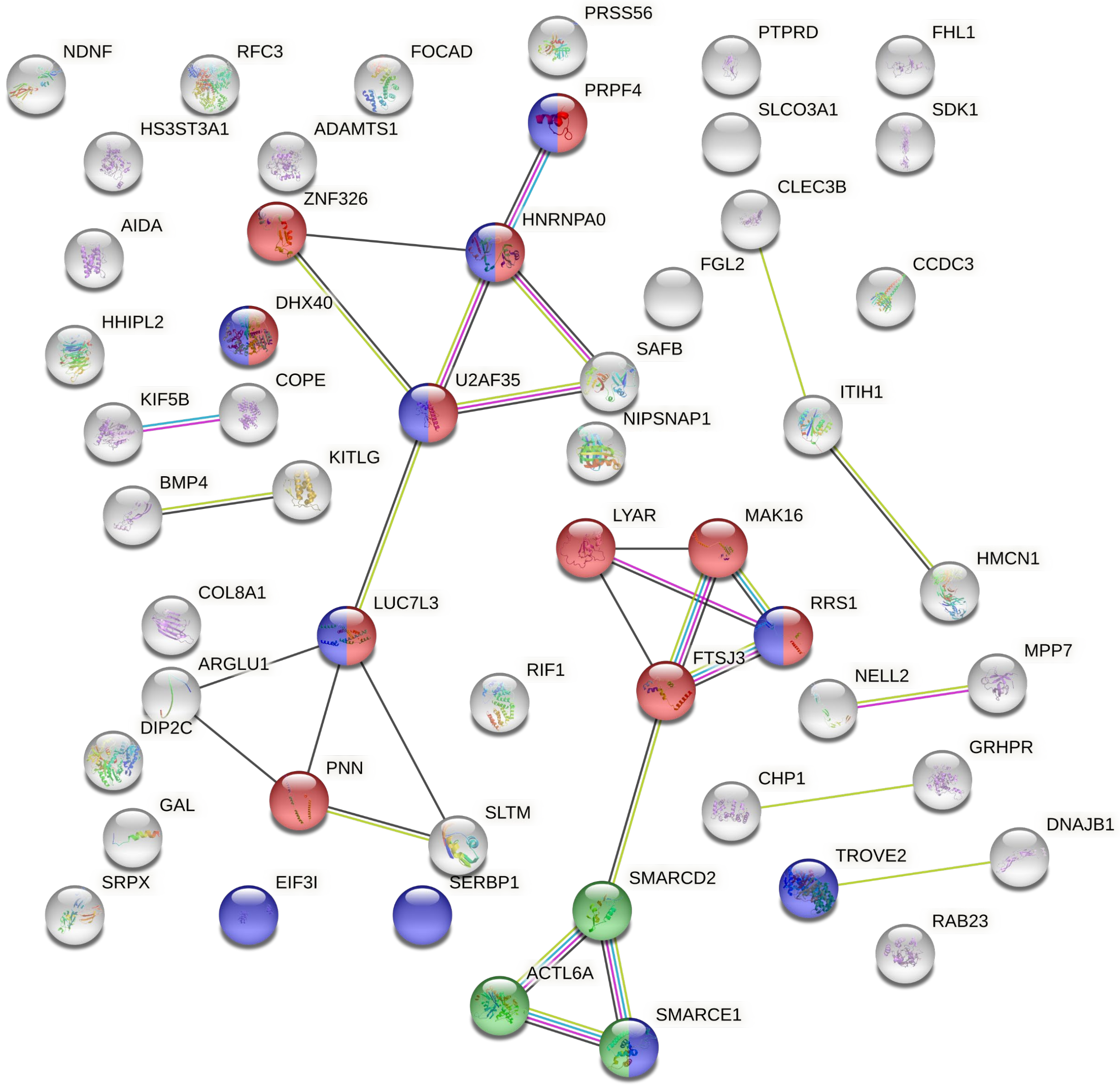

Supplementary Figure 6. STRING analysis of protein enrichment in EVs isolated from LK2 cell.

a Proteins exclusively presented in LK2-derived EVs (n = 11) were analysed by STRING database. 11 nodes/1 edge were identified. Nodes with top function enrichment are highlighted with red (Reactome Pathways, HAS-56511801, PCNA-Dependent Long Patch Base Excision Repair, strength 2.23).

b Proteins exclusively presented in EVs isolated from AZD4785-treated LK2 cells (n = 53) were analyzed by STRING database. 53 nodes/29 edges were identified. Nodes with top function enrichment are highlighted with red (Biological Process (Gene Ontology), GO:0006396, RNA processing, strength 0.69), blue (Molecular Function (Gene Ontology), GO:0003723, RNA binding, strength 0.64) and green (Cellular Component(Gene Ontology), GO:0016514, SWI/SNF complex, strength 1.87).

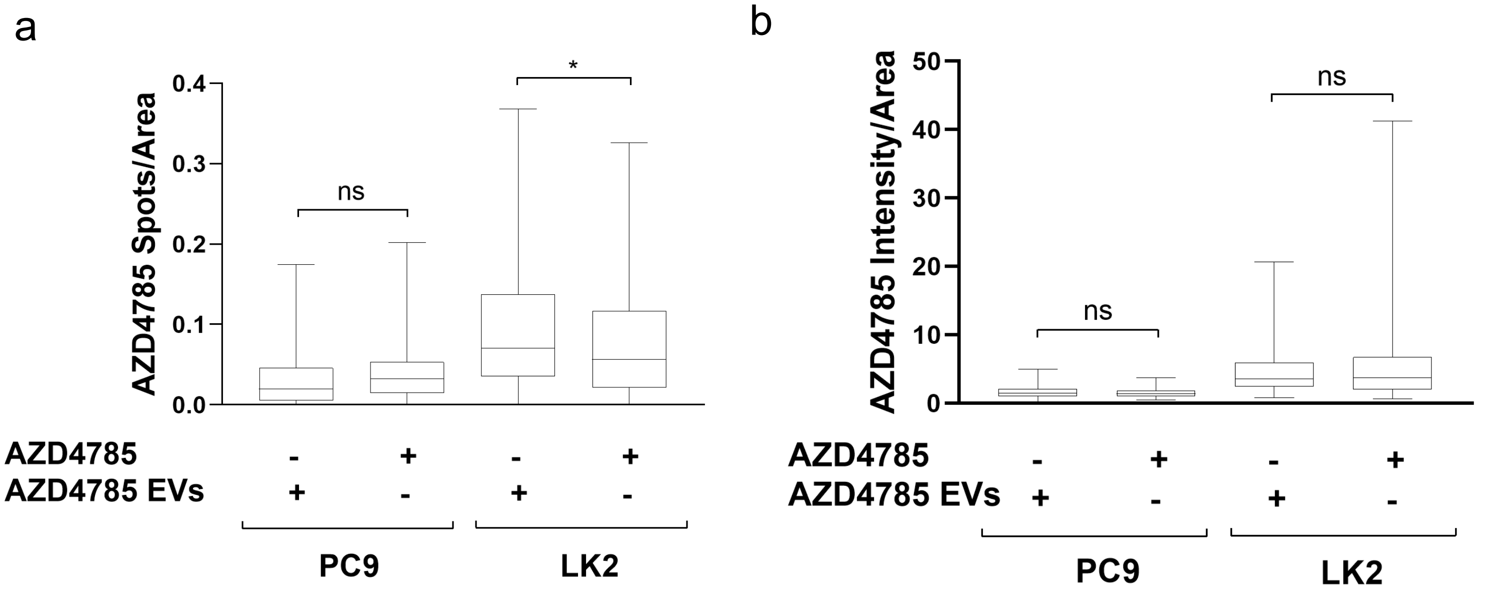

Supplementary Figure 7. Intracellular delivery of carrier-free and EV-loaded AZD4785. a, b Quantification of free and EV-loaded AZD4785 delivery to PC9 and LK2 cells. EVs were isolated from the conditioned media of the cells treated with 10μM AZD4785 for 24h as above. PC9 and LK2 cells were incubated with PC9- and LK2-derived AZD4785-loaded EVs, respectively, for 3h. Cells were washed, fixed and stained for CD63, AZD4785 and nucleus. Intracellular content of AZD4785 or overall intracellular intensity were quantified using Columbus software by counting number of stained loci in the cell cytosol ("Spots/Area", **a**) or average intensity ("Intensity/Area, **b**). Number of loci and intensity values were normalised to the cellular cytosol area. Free AZD4785 ("AZD4785") concentration was 250nM and AZD4785 dose in PC9 and LK2-derived EVs ("AZD4785 EVs") was 10nM and 20nM, correspondingly. Whiskers, Min to Max. Minimum 200 cells for each condition were counted (N=1).

a

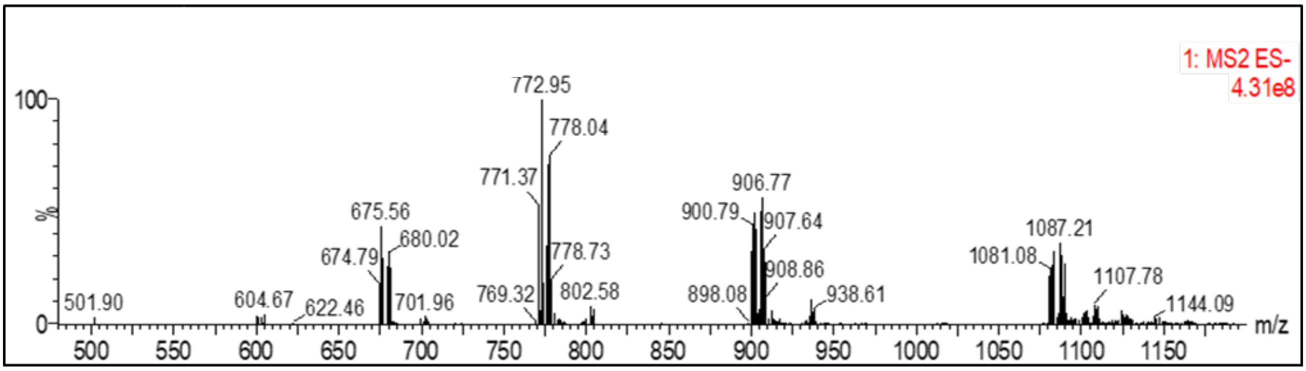

b

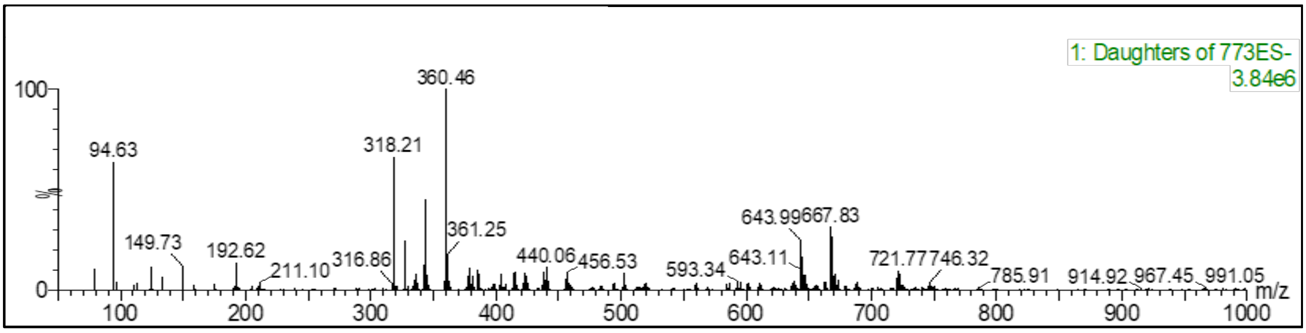

Supplementary Figure S8. Parent (**a**) and daughter (**b**) ion spectra from infusion of 10μM stock solution in DMSO.

a

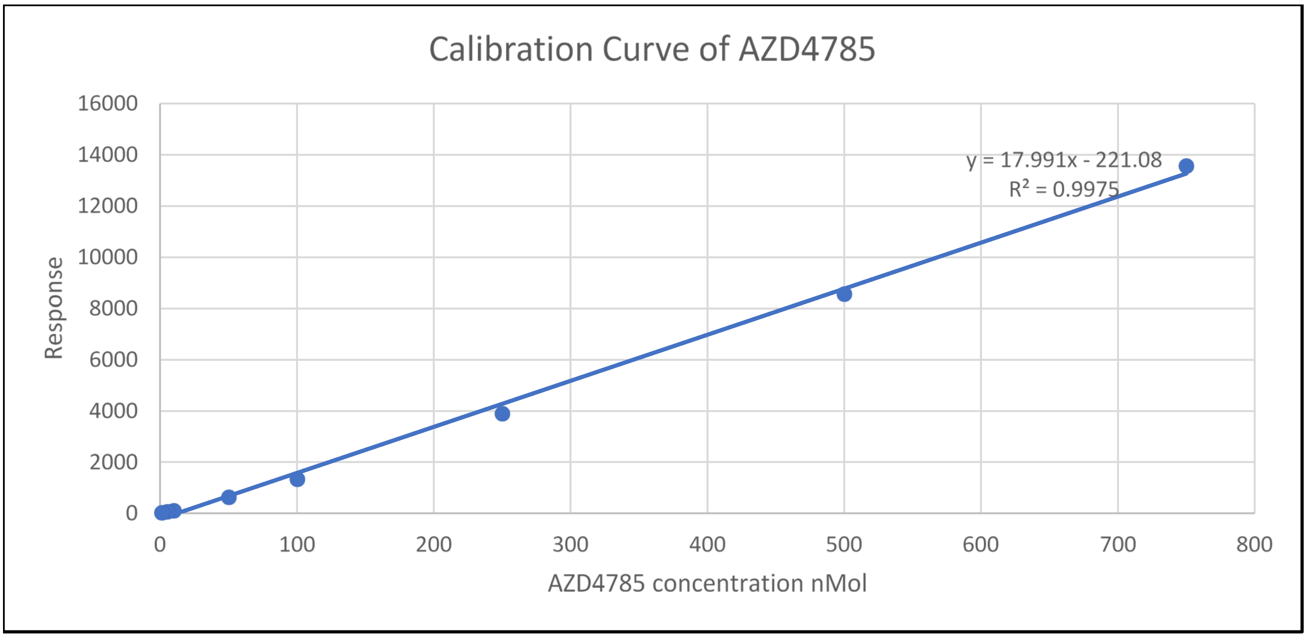

b

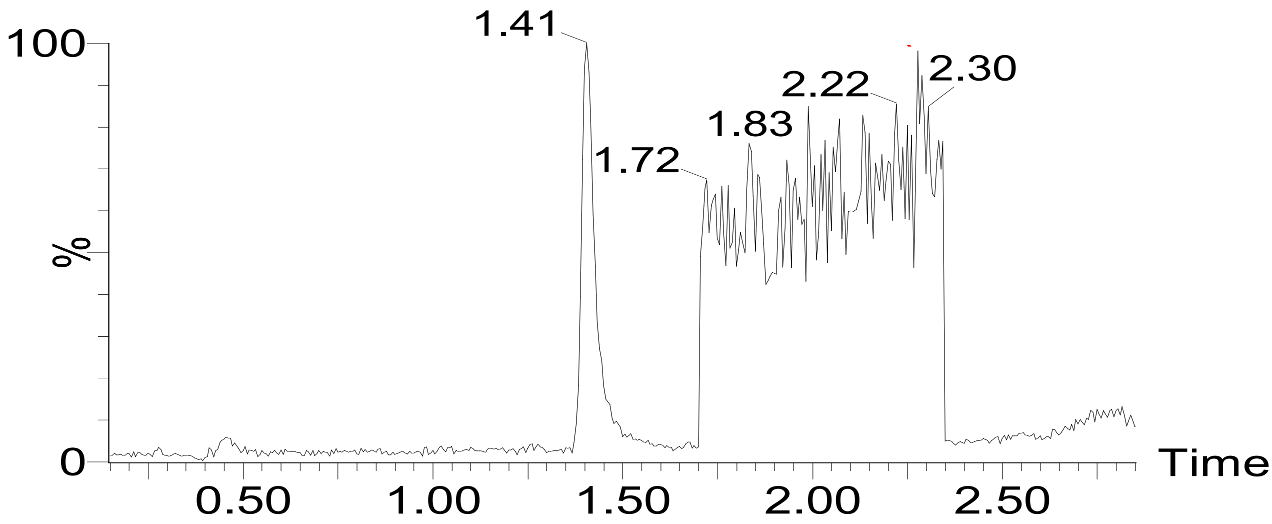

c

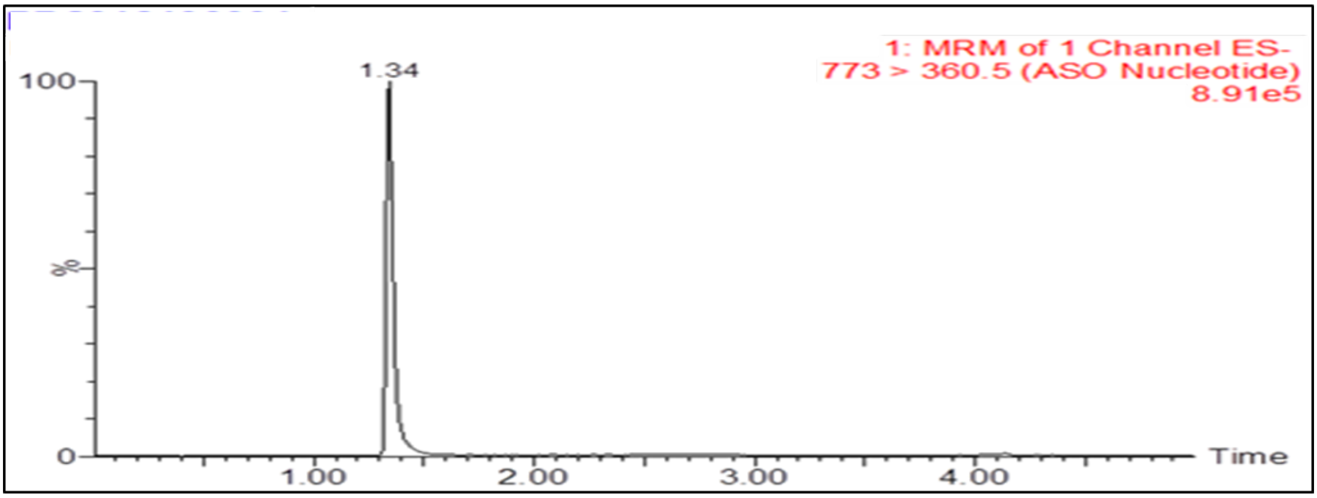

Supplementary Figure 9. a Representative standard curve of AZD4785 (as generated using TargetLynx™),
b Representative chromatograms showing S/N ratio of AZD4785 at 5 nM,
c Representative chromatography of AZD4785

a

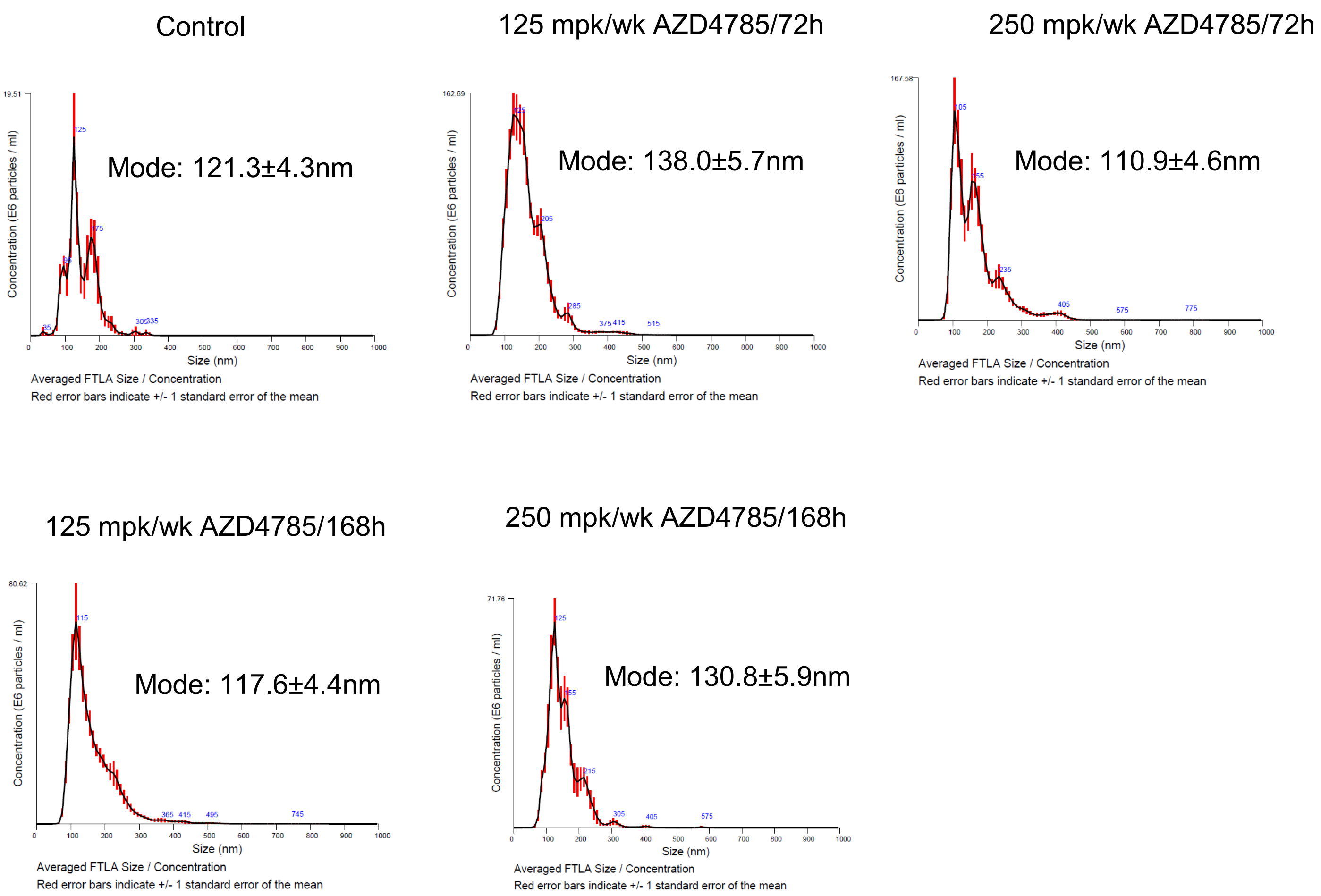

b

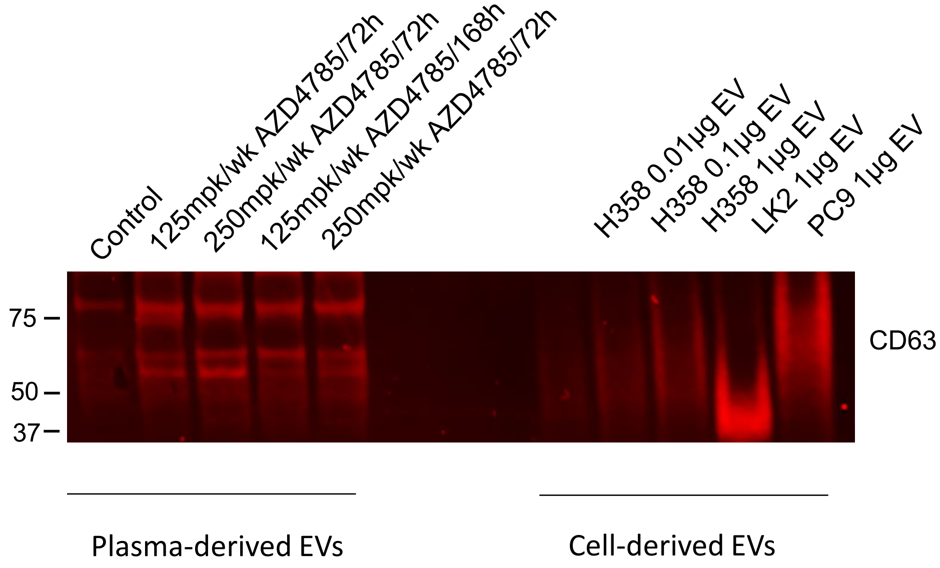

Supplementary figure S10. a Nanoparticle Tracking Analysis of EVs isolated from the plasma. PC9 xenograft mouse model. N = 5. **b** Western blotting analysis of EVs isolated from the plasma of PC9 xenograft mouse model. An aliquot of mouse plasma-derived EVs was separated by western blotting and stained for human CD63. Note that the CD63 is presented in EVs derived from the human tumour cell lines and it is heavily glycosylated and appear as a "smear" band on the western blot. However, the bands observed in the mice plasma-derived samples have different molecular weight and appearance indicating the absence of CD63 signal in plasma-derived samples.

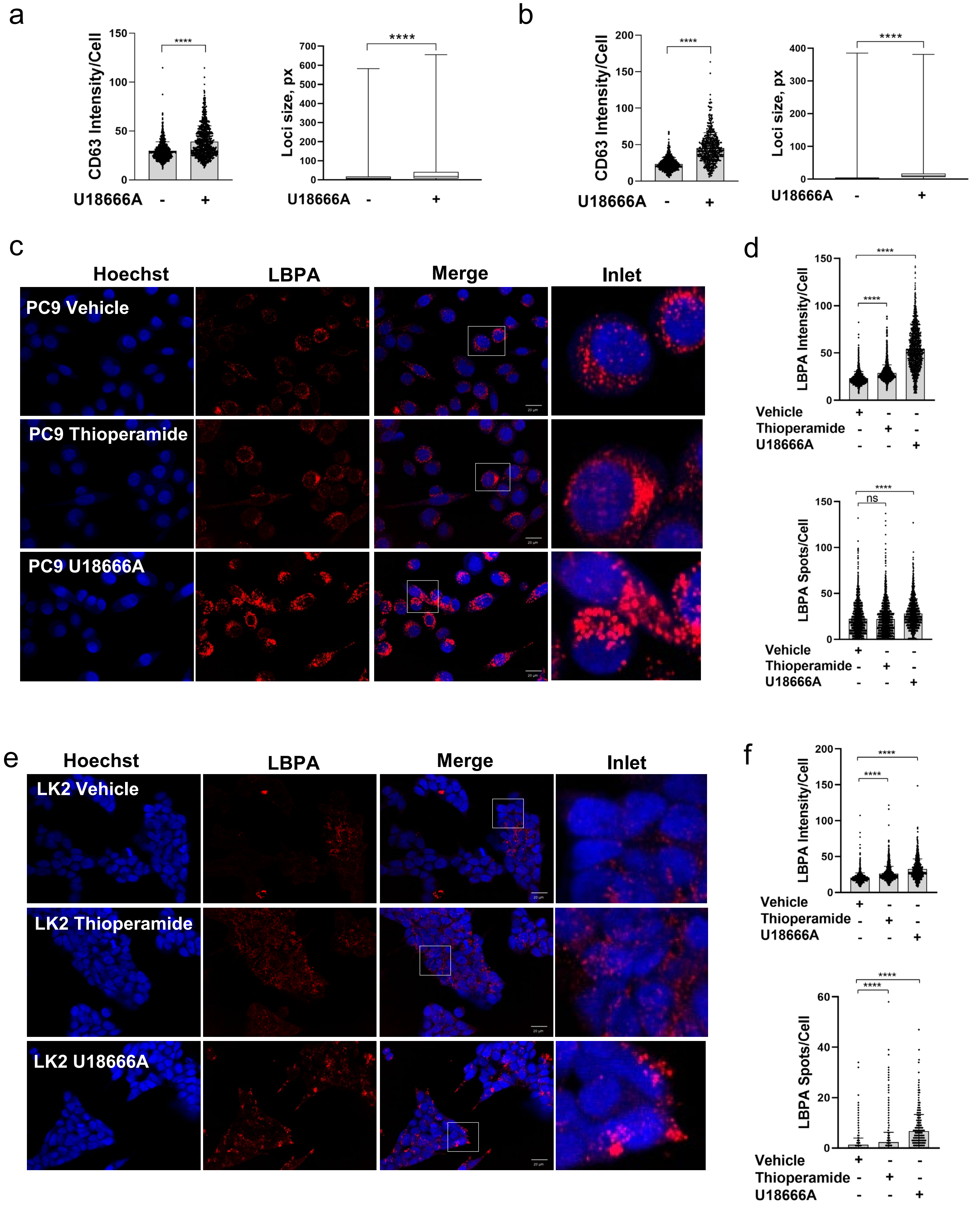

Supplementary Figure 11. Effect of thioperamide maleate and U18666A on intracellular levels of CD63 and LBPA.

PC9 (**a**, **c**, **d**) and LK2 (**b**, **e**, **f**) were fixed, permeabilised and stained with anti-CD63 and anti-LBPA antibodies, and visualized using secondary fluorescently-labelled antibodies. Cells were treated with 7.1μM U18666A or 10μM Thioperamide for 48h prior to fixation. Cell images were acquired using Opera microscope (60x NA 1.4 (**a**) or 40x NA 0.6. Error bars, Standard Deviation. Whiskers, Min to Max. TTEST ****, $p < 0.0001$ **c** Representative image of PC9 cells stained for LBPA **d** Quantification of intracellular content of CD63 and LBPA (“Intensity/Cell”) and number of spots (Spots/Cell) in single PC9 cells were measured by using Columbus software. Minimum 500 cells were measured for each conditions. Error bars, Standard Deviation. ANOVA ****, $p < 0.0001$. **, $p<0.01$. ns, non-significant. **e** Representative image of LK2 cells stained for LBPA **f** Quantification of intracellular content of CD63 and LBPA (“Intensity/Cell”) and number of spots (Spots/Cell) in single PC9 cells were measured by using Columbus software. Minimum 500 cells were measured for each conditions. Error bars, Standard Deviation. ANOVA ****, $p < 0.0001$. **, $p<0.01$. ns, non-significant.

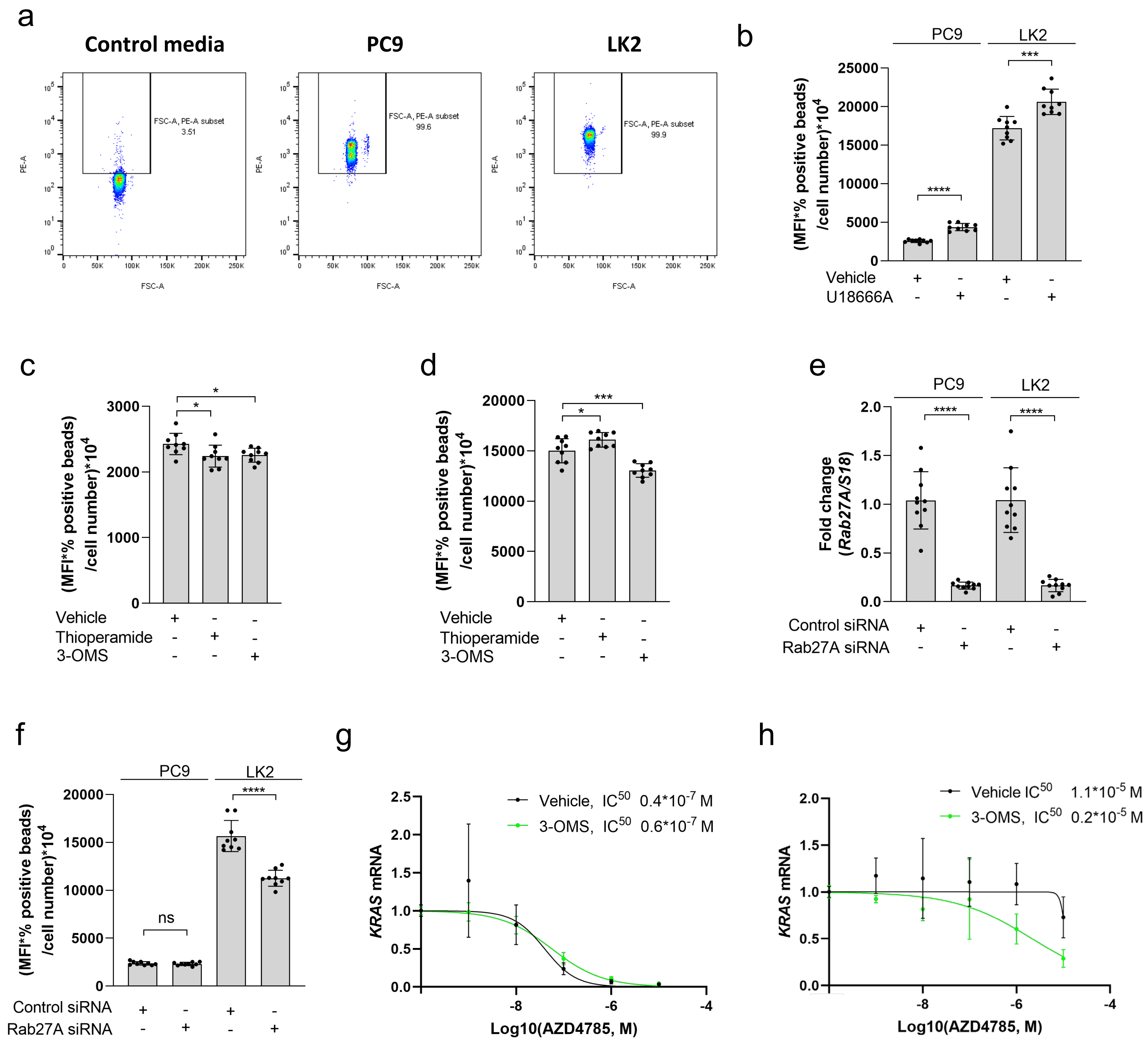

Supplementary Figure 12. Modulation of EV secretion by inhibitors and Rab27A siRNA.

a Representative flow cytometry dot-plot showing ant-CD81-PE staining after the treatment of CD63 capture beads either with the control cell culture media (not incubated with cells) or cell culture media incubated with PC9 and LK2 cells for 24h. Cell culture media – RMPI supplemented with 10% exosome-depleted FBS and GlutaMax. Percentages showing positive beads inside the boxed regions. N = 3. **b**-**d**, PC9 and LK2 cells were treated inhibitors for 24h in RPMI supplemented with 10% exosome-depleted FBS and GlutaMax and conditioned media was collected and incubated with CD63 capture beads and stained with anti-CD81-PE antibody. **b** Cells were treated with 7.1μM U18666A. PC9 (**c**) or LK2 (**d**) cells were treated with 10μM Thioperamide maleate or 10μM 3-O-Methyl-sphingomyelin (3-OMS). MFI, mean fluorescence intensity. Error bars, Standard Deviation, N=3, n=3 in each experiment. T-test, ****, P<0.0001, ***, P<0.001, *, P<0.05. **e** Rab27A expression in PC9 and LK2 cells after siRNA treatment. Cells were treated with control or Rab27A siRNA for 72h and Rab27A expression was quantified by using qPCR. Error bars, Standard Deviation, N=3, n=1-2. T-test, ****, P<0.0001. **f** EV secretion by PC9 and LK2 cells treated with siRNA for 48h in RPMI supplemented with 10% exosome-depleted FBS and GlutaMax and conditioned media was collected and incubated with CD63 capture beads and stained with anti-CD81-PE antibody. **g**, **h** *KRAS* expression by PC9 (**g**) and LK2 (**h**) cells after the AZD4785 treatment in the presence of 10μM 3-O-Methyl-sphingomyelin. Cells were treated with AZD4785 in RPMI supplemented with 10% exosome-depleted FBS and GlutaMax for 48h and *KRAS* expression was quantified by qPCR. Nonlinear regression analysis. Each data point are duplicates from N=3. Error bars, Standard Deviation.

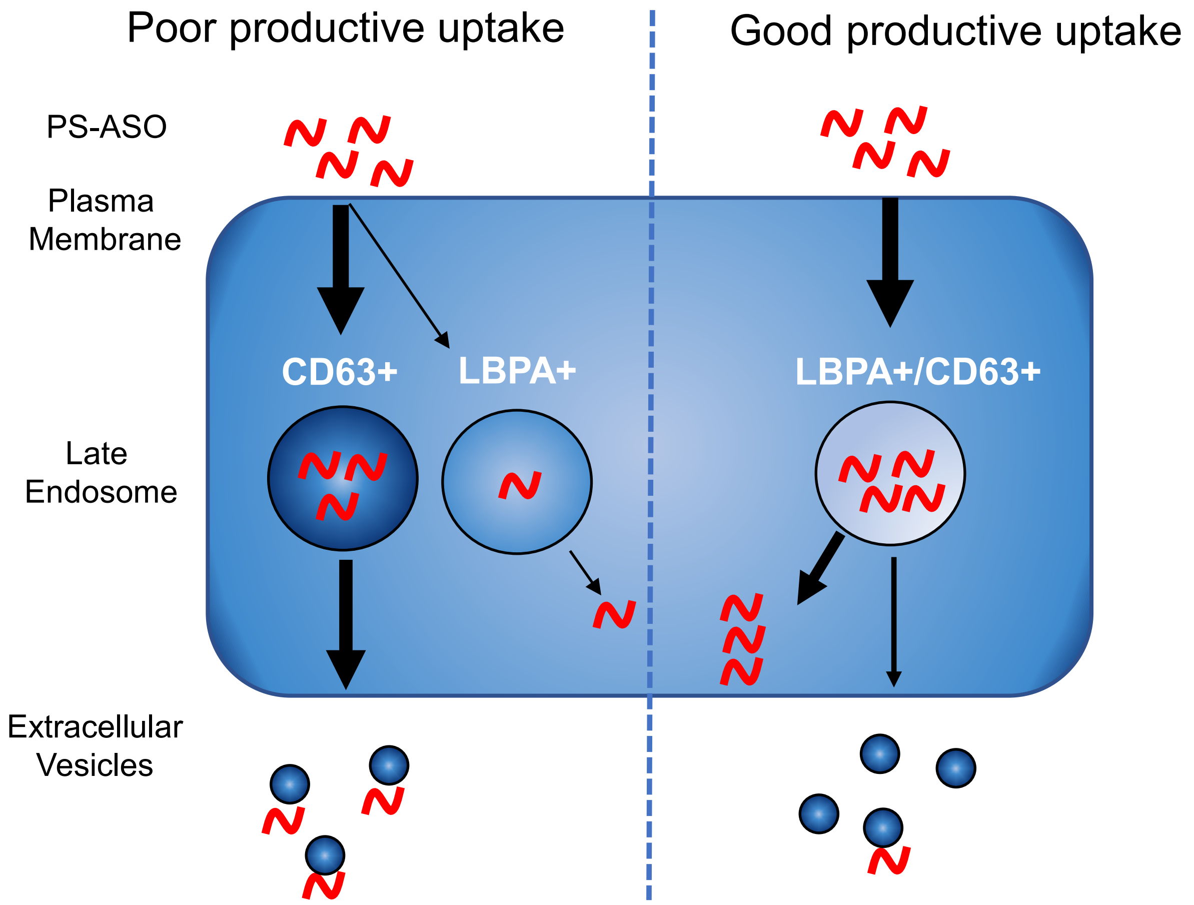

Supplementary Figure 13. Intracellular LBPA distribution and EV recycling pathway define good or poor productive uptake.

Antisense oligonucleotides are internalised by tumour cells and delivered to CD63+ late endosomes. Here, ASO escapes to cytosol with assistance of LBPA in the good productive uptake cells (PC9). In the poor productive uptake cells (LK2), LBPA is not presented in CD63 LE and ASO is secreted in extracellular vesicles.
