# Supplementary material for: Antisense oligonucleotide activity in tumour cells is influenced by intracellular LBPA distribution and extracellular vesicle recycling
Source: Commun Biol. 2021 Nov 1;4:1241. doi: 10.1038/s42003-021-02772-0 (PMC8560811; doi:10.1038/s42003-021-02772-0)
Supplement: Supplementary file 3 — Description of Additional Supplementary Files [file 42003_2021_2772_MOESM3_ESM.pdf]

Description of Additional Supplementary Files

File name: Supplementary Data 1
Description: Proteomic EV analysis.

File name: Supplementary Data 2
Description: Exlusive proteins in PC9 and LK2-derived EVs.

File name: Supplementary Data 3
Description: Raw data and western blots.
