# Supplementary material for: Antisense oligonucleotide activity in tumour cells is influenced by intracellular LBPA distribution and extracellular vesicle recycling
Source: Commun Biol. 2021 Nov 1;4:1241. doi: 10.1038/s42003-021-02772-0 (PMC8560811; doi:10.1038/s42003-021-02772-0)
Supplement: Supplementary file 7 — Reporting Summary [file 42003_2021_2772_MOESM7_ESM.pdf]

nature research

Corresponding author(s): Alexander Kapustin
COMMSBIO-19-1359B-Z

Last updated by author(s): Sep 21, 2021

Reporting Summary

Nature Research wishes to improve the reproducibility of the work that we publish. This form provides structure for consistency and transparency in reporting. For further information on Nature Research policies, see our Editorial Policies and the Editorial Policy Checklist.

Statistics

For all statistical analyses, confirm that the following items are present in the figure legend, table legend, main text, or Methods section.

n/a	Confirmed	
[ ]	[x]	The exact sample size (n) for each experimental group/condition, given as a discrete number and unit of measurement
[ ]	[x]	A statement on whether measurements were taken from distinct samples or whether the same sample was measured repeatedly
[ ]	[x]	The statistical test(s) used AND whether they are one- or two-sided<br>*Only common tests should be described solely by name; describe more complex techniques in the Methods section.*
[x]	[ ]	A description of all covariates tested
[x]	[ ]	A description of any assumptions or corrections, such as tests of normality and adjustment for multiple comparisons
[ ]	[x]	A full description of the statistical parameters including central tendency (e.g. means) or other basic estimates (e.g. regression coefficient) AND variation (e.g. standard deviation) or associated estimates of uncertainty (e.g. confidence intervals)
[ ]	[x]	For null hypothesis testing, the test statistic (e.g. F, t, r) with confidence intervals, effect sizes, degrees of freedom and P value noted<br>*Give P values as exact values whenever suitable.*
[x]	[ ]	For Bayesian analysis, information on the choice of priors and Markov chain Monte Carlo settings
[x]	[ ]	For hierarchical and complex designs, identification of the appropriate level for tests and full reporting of outcomes
[x]	[ ]	Estimates of effect sizes (e.g. Cohen's d, Pearson's r), indicating how they were calculated

Our web collection on statistics for biologists contains articles on many of the points above.

Software and code

Policy information about availability of computer code

Data collection	No software was used for data collection.
Data analysis	Image Analysis was conducted by using Columbus (PerkinElmer, Columbus 2.8.3.1266).<br>NTA analysis was performed by using NanoSight NTA software v3.2 (Malvern, Worcestershire, United Kingdom).<br>Western blots were quantified using Odyssey CLx Infrared Imaging System software.<br>Statistical analysis was performed with GraphPad PRISM (version 8.0.1(244)) software.

For manuscripts utilizing custom algorithms or software that are central to the research but not yet described in published literature, software must be made available to editors and reviewers. We strongly encourage code deposition in a community repository (e.g. GitHub). See the Nature Research guidelines for submitting code & software for further information.

Data

Policy information about availability of data

All manuscripts must include a data availability statement. This statement should provide the following information, where applicable:

- Accession codes, unique identifiers, or web links for publicly available datasets
- A list of figures that have associated raw data
- A description of any restrictions on data availability

The authors declare that all relevant data supporting the findings of the study are available within the manuscript and its supporting information.
The mass spectrometry proteomics data have been deposited to the ProteomeXchange Consortium via the PRIDE partner repository with the dataset identifier PXD027804. Unedited western blot images are included in the Supplementary Data. All other data for all figures and results presented here are available from the corresponding author (ANK) upon reasonable request.

Field-specific reporting

Please select the one below that is the best fit for your research. If you are not sure, read the appropriate sections before making your selection.

☒ Life sciences ☐ Behavioural & social sciences ☐ Ecological, evolutionary & environmental sciences

For a reference copy of the document with all sections, see nature.com/documents/nr-reporting-summary-flat.pdf

Life sciences study design

All studies must disclose on these points even when the disclosure is negative.

Sample size	For in vitro experiments at least 3 independent experiments were conducted with technical replicates. For experiments in vivo a minimum of 5 animals per treatment group was used. Number of animals was estimated from the previous study of AZD4785 effects in mice xenograft model (Sarah J. Ross et al., Science Translational Medicine 14 Jun 2017: Vol. 9, Issue 394).
Data exclusions	No data were excluded from the analysis.
Replication	All experiments were measured in technical replicates, with a minimum of three biological replicates (N=3). All attempts to replication of the described findings were successful.
Randomization	Age-matched animals were randomly assigned into treatment groups (n = 5) to ensure an equal tumor size across groups at the initiation of the study.
Blinding	Samples for mass spectrometry analysis were assigned experimental identifier number and data analysis was conducted by individuals blinded to the assigned experimental conditions of the sample being analyzed. Other in vitro and in vivo experiments were not performed blinded.

Reporting for specific materials, systems and methods

We require information from authors about some types of materials, experimental systems and methods used in many studies. Here, indicate whether each material, system or method listed is relevant to your study. If you are not sure if a list item applies to your research, read the appropriate section before selecting a response.

Materials & experimental systems

n/a	Involved in the study	
☐	☒	Antibodies
☐	☒	Eukaryotic cell lines
☒	☐	Palaeontology and archaeology
☐	☒	Animals and other organisms
☒	☐	Human research participants
☒	☐	Clinical data
☒	☐	Dual use research of concern

Methods

n/a	Involved in the study	
☒	☐	ChIP-seq
☐	☒	Flow cytometry
☒	☐	MRI-based neuroimaging

Antibodies

Antibodies used

Bovine Serum Albumin antibody [EPR12774] (Abcam, ab192603)
Mouse Monoclonal antibody [clone 2C1] (IgG2b) to Human KRAS (LSBio LifeSpan Bioscience Inc, LSB LS-C175665)
Vinculin antibody (Abcam, ab73412)
Human CD63 antibody (BD Pharmingen 556019)
Alix Antibody (Thermo Fisher Scientific, MA1-83977)
LBPA Antibody (Clone 6C4, MABT837, Merck)
Rabbit anti-ASO antibody (Ionis 13545)
Secondary Donkey Anti-Rabbit IRDye@800 CW (Li-COR 926-32213)
Secondary donkey anti-mouse IRDye@680 LT (Li-COR 926-68022)
Donkey anti-Rabbit IgG (H+L) ,Alexa Fluor 568 (Thermo Fisher Scientific, A10042)
Donkey anti-Mouse IgG (H+L), Alexa Fluor 488 (Thermo Fisher Scientific, A21202)
Goat anti-Rabbit IgG(H+L), Alexa Fluor488 (Thermo Fisher Scientific, A11008)
Goat anti-Mouse IgG (H+L) Highly Cross-Adsorbed Secondary Antibody, Alexa Fluor 647 (Thermo Fisher Scientific, A21236)

Validation

Bovine Serum Albumin antibody [EPR12774] (Abcam, ab192603) application validated by manufacturer: https://www.abcam.com/bovine-serum-albumin-antibody-epr12774-ab192603.html?productWallTab=ShowAll
Mouse Monoclonal antibody [clone 2C1] (IgG2b) to Human KRAS (LSBio LifeSpan Bioscience Inc, LSB LS-C175665) Validated by

April 2020

manufacturer: https://www.lsbio.com/antibodies/kras-antibody-clone-2c1-ihc-wb-western-ls-c175665/183089#validation-section
Vinculin antibody (Abcam, ab73412). Application is validated by the manufacturer: https://www.abcam.com/vinculin-antibody-ab73412.html
Human CD63 antibody (BD Pharmingen 556019) Validated by the manufacturer: https://www.bdbiosciences.com/us/reagents/research/antibodies-buffers/immunology-reagents/anti-human-antibodies/cell-surface-antigens/purified-mouse-anti-human-cd63-h5c6/p/556019
Alix Antibody (Thermo Fisher Scientific, MA1-83977), Application validated by the manufacturer https://www.thermofisher.com/antibody/product/Alix-Antibody-clone-3A9-Monoclonal/MA1-83977
LBPA Antibody (Clone 6C4, MABT837, Merck) use for immunocytochemistry validated by manufacturer https://www.merckmillipore.com/GB/en/product/Anti-LBPA-Antibody-clone-6C4,MM_NF-MABT837#anchor_REF
rabbit anti-ASO (Ionis 13545), Application of this antibody for cytochemistry was validated before: https://www.ncbi.nlm.nih.gov/pmc/articles/PMC6511877/
Secondary Donkey Anti-Rabbit IRDye@800 CW (Li-COR 926-32213)
Antibody use was validated before: https://www.licor.com/bio/reagents/irdye-800cw-donkey-anti-rabbit-igg-secondary-antibody
Secondary donkey anti-mouse IRDye@680 LT (Li-COR 926-68022)
https://www.licor.com/bio/reagents/irdye-680lt-donkey-anti-mouse-igg-secondary-antibody
Donkey anti-Rabbit IgG (H+L), Alexa Fluor 568 (A10042); application validated by manufacturer: https://www.thermofisher.com/antibody/product/Donkey-anti-Rabbit-IgG-H-L-Highly-Cross-Adsorbed-Secondary-Antibody-Polyclonal/A10042
Donkey anti-Mouse IgG (H+L), Alexa Fluor 488 (A21202); application validated by the manufacturer:
https://www.thermofisher.com/antibody/product/Donkey-anti-Mouse-IgG-H-L-Highly-Cross-Adsorbed-Secondary-Antibody-Polyclonal/A-21202
Goat anti-Mouse IgG (H+L), Alexa Fluor 647 (A21236); application validated by the manufacturer:
https://www.thermofisher.com/antibody/product/Goat-anti-Mouse-IgG-H-L-Highly-Cross-Adsorbed-Secondary-Antibody-Polyclonal/A-21236
Goat anti-Rabbit IgG(H+L), Alexa Fluor488 (Thermo Fisher Scientific, A11008) use for immunocytochemistry validated by the manufacturer: https://www.thermofisher.com/antibody/product/Goat-anti-Rabbit-IgG-H-L-Cross-Adsorbed-Secondary-Antibody-Polyclonal/A-11008
Goat anti-Mouse IgG (H+L) Highly Cross-Adsorbed Secondary Antibody, Alexa Fluor 647 (Thermo Fisher Scientific, A21236) application validated by the manufacturer: https://www.thermofisher.com/antibody/product/Goat-anti-Mouse-IgG-H-L-Highly-Cross-Adsorbed-Secondary-Antibody-Polyclonal/A-21236

Eukaryotic cell lines

Policy information about cell lines

Cell line source(s)	PC9 (formerly known as PC-14): ECACC General Cell Collection: (https://www.pheculturecollections.org.uk/products/celllines/generalcell/detail.jsp?refId=900718103 &collection=ecacc_gc). LK2 cell: Japanese Collection of Research Bioresources Cell 4 Bank (https://cellbank.nibiohn.go.jp/~cellbank/en/search_res_det.cgi?ID=540).
Authentication	PC9 and LK2 cell lines were authenticated by short tandem repeat (STR) analysis.
Mycoplasma contamination	Both cell lines were negative for mycoplasma contamination.
Commonly misidentified lines (See ICLAC register)	Both cell lines are not in the ICLAC register (Version 9, released 14 October 2018).

Animals and other organisms

Policy information about studies involving animals; ARRIVE guidelines recommended for reporting animal research

Laboratory animals	Female SCID mice (Envigo) were housed under specific pathogen-free conditions in individually ventilated cages (Techniplast) at Alderley Park, United Kingdom. Mice had access to water and food ad libitum. Study was conducted in 8- to 12- week old female mice in full accordance with the UK Home Office Animal (Scientific Procedures) Act 1986.
Wild animals	No wild animals were used for this study.
Field-collected samples	No field-collected samples were collected for this study.
Ethics oversight	All animal experiments were approved by AstraZeneca animal welfare ethical review board.

Note that full information on the approval of the study protocol must also be provided in the manuscript.

Flow Cytometry

Plots

Confirm that:

- [x] The axis labels state the marker and fluorochrome used (e.g. CD4-FITC).
- [x] The axis scales are clearly visible. Include numbers along axes only for bottom left plot of group (a 'group' is an analysis of identical markers).
- [ ] All plots are contour plots with outliers or pseudocolor plots.
- [x] A numerical value for number of cells or percentage (with statistics) is provided.

Methodology

Sample preparation	CD63 capture beads were treated with conditioned cell media and stained with anti-CD81-PE antibody. Detailed protocol is provided in the Methods.
Instrument	LSR Fortessa, Beckton Dickinson, San Jose, CA
Software	FlowJo 10.7.1
Cell population abundance	cells were not analyzed
Gating strategy	Population of single beads was gated using FlowJo 10.7.1

- [x] Tick this box to confirm that a figure exemplifying the gating strategy is provided in the Supplementary Information.
